# Continuation of anti-PD-1 therapy plus physician-choice treatment beyond first progression is not associated with clinical benefit in patients with advanced non-small cell lung cancer

**DOI:** 10.3389/fimmu.2023.1151385

**Published:** 2023-05-29

**Authors:** Yixing Wang, Sha Fu, Xuanye Zhang, Wei Du, Linfeng Luo, Yongluo Jiang, Yixin Zhou, Yuanyuan Zhao, Yunpeng Yang, Hongyun Zhao, Wenfeng Fang, Yan Huang, Li Zhang, Shaodong Hong

**Affiliations:** ^1^ State Key Laboratory of Oncology in South China, Guangzhou, China; ^2^ Collaborative Innovation Center for Cancer Medicine, Guangzhou, China; ^3^ Department of Medical Oncology, Sun Yat-sen University Cancer Center, Guangzhou, China; ^4^ Department of Cellular & Molecular Diagnostics Center, Sun Yat-Sen Memorial Hospital, Sun Yat-Sen University, Guangzhou, China; ^5^ Guangdong Provincial Key Laboratory of Malignant Tumor Epigenetics and Gene Regulation of Sun Yat-Sen University, Guangzhou, China; ^6^ Department of Nuclear Medicine, Sun Yat-sen University Cancer Center, Guangzhou, China; ^7^ Department of VIP Region, Sun Yat-sen University Cancer Center, Guangzhou, China; ^8^ Department of Clinical Research, Sun Yat-sen University Cancer Center, Guangzhou, China

**Keywords:** immune checkpoint inhibitors, disease progression, second-line therapy, non-small-cell lung cancer, clinical benefit

## Abstract

**Objective:**

Few data are available on the optimal treatment options after disease progression from first-line treatment of immune checkpoint inhibitors (ICIs) plus chemotherapy. This study aimed to describe the safety and efficacy of continuing ICIs beyond first progress disease (PD) in non-small cell lung cancer (NSCLC).

**Methods:**

Patients with NSCLC previously treated with first-line anti-PD-1 antibody plus platinum-doublet chemotherapy and hence had PD as per Response Evaluation Criteria in Solid Tumors v1.1 were enrolled. For the subsequent line, patients received physician’s choice (PsC) with or without an anti-PD-1 antibody. The primary outcome was progression-free survival after second-line treatment (PFS2). Secondary outcomes included overall survival (OS) from the initiation of first-line treatment, post-second-progression survival (P2PS), overall response rate (ORR), disease control rate (DCR), and safety during second-line treatment.

**Results:**

Between July 2018 and January 2021, 59 patients were included. A total of 33 patients received a physician-decided second-line regimen plus ICIs (PsC plus ICIs group), and 26 patients did not continue ICIs (PsC group). There was no significant difference in PFS2 between the PsC plus ICIs group and the PsC group (median, 6.5 vs. 5.7 months, *p* = 0.46). median OS (28.8 vs. 29.2 months), P2PS (13.4 vs. 18.7 months), ORR (18.2% vs. 19.2%), and DCR (78.8% vs, 84.6%) were also similar between the two groups. No new safety signals were observed.

**Conclusion:**

In this real-world setting, patients treated with continued ICIs beyond their first disease progression did not experience clinical benefit but without compromising safety.

## Introduction

In recent years, the introduction of immune checkpoint inhibitors (ICIs), including anti-programmed cell death 1 (PD-1) or anti-programmed cell death ligand 1 (PD-L1) therapies, has represented a major advance in the treatment of advanced non-small cell lung cancer (NSCLC), allowing sustained recovery and disease remission in a significant proportion of patients (1, 2). Among advanced NSCLC without driver alterations, the available drugs are classified into three therapeutic classes: cytotoxic agents (e.g., pemetrexed, albumin paclitaxel, cisplatin, carboplatin, gemcitabine, and S-1), angiogenesis inhibitors (e.g., bevacizumab and anlotinib), and immunotherapy (e.g., anti-PD-1, pembrolizumab, tislelizumab, nivolumab, camrelizumab, sintilimab, toropalimab, anti-PD-L1, and atezolizumab) (3, 4).

Randomized trials have revealed that anti-PD-1/PD-L1 plus platinum-based chemotherapy (PBC) provides additional benefits in both overall survival (OS) and progression-free survival (PFS) for patients with advanced NSCLC, compared with chemotherapy alone in first-line treatment (5–10). Furthermore, many trials demonstrated the clinical benefits and favorable tolerability of anti-PD-1/PD-L1 for previously treated with chemotherapy in NSCLC patients (11–14). This raises the question of what is the most appropriate second-line treatment option after PD-1 treatment beyond response evaluation criteria in solid tumors (RECIST) v1.1 defined progress disease (PD).

In standard chemotherapy, because disease progression is assimilated with the development of drug resistance, guidelines recommend switching to a different agent in the second line. However, this dogma has been challenged in the times of immunotherapy due to the limited understanding of mechanisms of resistance to ICIs (15). Moreover, the potential OS benefit of ICIs was not always captured by RECIST v1.1, which are more appropriate surrogate endpoints for assessing the survival benefit of chemotherapy (16). Several evaluation criteria such as immune-related RECIST (irRECIST) and modified RECIST1.1 for immune-based therapeutics (iRECIST) have been proposed by the RECIST working group in cancer immunotherapy trials (17). However, it is still unknown whether the possibility of delayed, immune-related responses may suggest that ICIs could be beneficial for patients with disease progression.

Some studies have suggested that continuation of ICIs in second-line treatment may be beneficial in advanced melanoma and metastatic renal cell carcinoma (18, 19). However, only a few studies have examined whether continuing ICIs beyond progress of disease is safe and effective for patients with advanced NSCLC. The objective of this retrospective analysis was to describe the effectiveness of continued ICIs plus physician’s choice (PsC) beyond the first progression in NSCLC in the real-world setting.

## Materials and methods

### Patients

We performed a retrospective cohort study of continued ICIs plus PsC after the first progression in advanced NSCLC. This study involved consecutive patients referred to Sun Yat-sen University Cancer Center between 1 July 2018 and 31 January 2021. Patients were included in the study if they met the following criteria: (1) pathologically confirmed NSCLC and without driver alterations; (2) received at least one cycle of immunotherapy combined with platinum-based doublet chemotherapy in the first line and defined PD at the end of the first line; and (3) complete clinicopathological data for evaluation. In the second line, all medications, including immunotherapy, antiangiogenic therapy, and chemotherapy, were acceptable.

The study protocol conforms to the ethical guidelines of the Declaration of Helsinki as reflected in *a priori* approval by the Sun Yat-Sen University Cancer Center Institutional Review Board. Since this study was retrospectively designed, informed consent was waived.

### Data collection

Patients’ clinicopathologic features and treatment were retrospectively collected from electronic medical records. We evaluated baseline characteristics, including sex, age, pathology, Eastern Cooperative Oncology Group (ECOG) performance status, clinical stage, and presence of metastatic sites. Clinical outcomes include the overall response rate (ORR), disease control rate (DCR), PFS (PFS1, PFS2, PFS1 + 2), post-second-progression survival (P2PS), and OS. The ORR was defined as the proportion of patients with the best overall response of complete response (CR) or partial response (PR). The DCR was defined as the proportion of patients with the best overall response of CR or PR or stable disease (SD). PFS1 was defined as the time of initiation of immunotherapy combined with chemotherapy to first RECIST 1.1–defined PD. PFS2 was the time from the first defined PD to the second disease progression or the next-line systemic therapy or death. PFS1 + 2 was defined as the time of initiation of immunotherapy combined with chemotherapy to the second disease progression or the next-line systemic therapy or death. OS was defined as the date of commencing immunotherapy combined with chemotherapy treatment to death. The timespan between the first progression and death/last follow-up was defined as P2PS.

Safety evaluation was conducted in all eligible patients. Adverse events were graded for severity using the National Cancer Institute Common Terminology Criteria for Adverse Events, version 4.0.

### Statistical analysis

Differences in baseline between groups were compared using chi-square or Fisher’s exact test for categorical data and the Wilcoxon rank-sum test for continuous data. The median PFS and the median OS (with their 95% CIs) were determined using the Kaplan–Meier method. Hazard ratios (HRs) and their 95% CIs were calculated by using Cox proportional hazards regression models to assess the effects of different variables on PFS and OS. The median follow-up times and their 95% CIs were determined using the reverse Kaplan–Meier method. A two-tailed *P* value of ≤0.05 defined statistical significance. All statistical analyses were performed using the R software version 4.2.0 (https://www.r-project.org/).

## Results

### Patient characteristics

From 1 June 1 2018 to 30 June 2021, 59 patients were enrolled in this study. The baseline demographic and clinical characteristics are summarized in **Table 1**. The median age was 60 years (range, 31–74), 51 were men (86.4%), 34 were smokers (57.6%), 34 have non-squamous cell carcinoma (57.6%), and 51 were in stage IV (86.4%). All patients have received chemoimmunotherapy and were finally evaluated as PD in first-line treatment. The anti-PD-1 drugs used in the study were pembrolizumab (26, 44.1%), nivolumab (1, 1.7%), sintilimab (19, 32.2%), tislelizumab (1, 1.7%), camrelizumab (7, 11.9%), and toropalimab (5, 8.4%). As per RECIST 1.1, 28 (47.4%) patients achieved PR as best overall response, 27 (45.8%) achieved SD, and 4 (57.5%) had PD. The ORR was 47.4%, and the DCR was 93.2%. The median PFS1 was 7.95 months (95% confidence interval [CI], 6.396–9.505).

**Table 1:** Patient characteristics at baseline.

Characteristics (N = 59)	NO. (%)
Age	
Median (range)	60 (31–74)
<60	27 (45.8)
≥60	32 (54.2)
Sex	
Female	8 (13.8)
Male	51 (86.4)
ECOG performance	
0	39 (66.1)
1	15 (25.4)
2–3	5 (8.5)
Smoking status	
Never smoker	25(42.4)
Current or former smoker	34 (57.6)
Pathology	
Squamous cell carcinoma	25 (42.4)
Non-squamous cell carcinoma	34 (57.6)
Stage	
IIIC	8 (13.6)
IV	51 (86.4)
Metastatic sites	
Liver	6 (10.2)
Brain	8 (13.6)
Bone	13 (22.0)
Intrapulmonary	14 (23.7)
Adrenal glands	4 (6.8)
No. of metastatic organs	
0–1	41 (69.5)
≥2	18 (30.5)
Type of ICIs	
Pembrolizumab	26 (44.1)
Tislelizumab	1 (1.7)
Nivolumab	1 (1.7)
Camrelizumab	7 (11.9)
Sintilimab	19 (32.2)
Toropalimab	5 (8.4)
First-line progression-free survival, mo	
<8	30 (50.8)
≥8	29 (49.2)
RECIST response in the first line	
PR	28 (47.4)
SD	27 (45.8)
PD	4 (6.8)

ECOG, Eastern Cooperative Oncology Group; RECIST, Response Evaluation Criteria in Solid Tumors.

In the second-line treatment, the detailed treatment is shown in **Table S1**. Most patients continue platinum-based doublet chemotherapy plus ICIs (DC plus ICIs, 17, 28.8%). Three patients received antiangiogenic (A), two patients received antiangiogenic plus ICIs (A plus ICIs), nine patients received double-agent chemotherapy (DC), nine patients received double-agent chemotherapy plus antiangiogenic (DC plus A), five patients received double-agent chemotherapy+ antiangiogenic plus ICIs (DC plus A plus ICIs), four patients received single-agent chemotherapy (SC), three patients received single-agent chemotherapy plus antiangiogenic (SC plus A), four patients received single-agent chemotherapy plus antiangiogenic plus ICIs (SC plus A plus ICIs), and four patients received single-agent chemotherapy plus ICIs (SC plus ICIs).

Patient characteristics between the PsC plus ICIs group and the PsC group are shown in **Table 2**. A total of 33 patients (55.9%) continued to use PsC plus ICIs therapy as second-line treatment. All baseline characteristics, including age, sex, ECOG performance, smoking status, pathology, stage, the number of metastatic sites, PFS1, and treatment in the second line, were relatively comparable between the PsC plus ICIs group and PsC groups.

**Table 2:** Baseline characteristics of the included patients treated with or without immune checkpoint inhibitors.

Characteristics	PsC plus ICIs group (N = 33)	PsC group (N = 26)	*P*
**Age**			0.546
Median (range)	60 (31-74)	61 (31-72)	
**Sex**			0.446
Female	3 (9.1)	5 (19.2)	
Male	30 (90.9)	21 (80.8)	
**ECOG performance**			0.774
0	23 (69.7)	16 (61.5)	
1	7 (21.2)	8 (30.8)	
2–3	3 (9.1)	2 (7.7)	
**Smoking status**			0.791
Never smoker	13 (39.4)	12 (46.2)	
Current or former smoker	20 (60.6)	14 (53.8)	
**Pathology**			0.791
Squamous cell carcinoma	13 (39.4)	12 (46.2)	
Non-squamous cell carcinoma	20 (60.6)	14 (53.8)	
**Stage**			1.000
IIIC	4 (12.1)	4 (15.4)	
IV	29 (87.9)	22 (84.6)	
**No. of metastatic sites**			1.000
0–1	23 (69.7)	18 (69.2)	
≥2	10 (33.3)	8 (30.8)	
**First-line progression-free survival, mo**			0.726
PFS1	7.55 months (95% CI, 6.004–9.109)	8.08 months (95% CI, 5.193–10.972)	
**RECIST response in the first line**			0.088
PR	13 (39.4)	15 (57.7)	
SD	19 (57.6)	8 (30.8)	
PD	1 (3.0)	3 (11.5)	
**Treatment in the second line**			0.571
Single-agent chemotherapy	4 (12.1)	4 (15.4)	
Double-agent chemotherapy	17 (51.5)	8 (30.8)	
Antiangiogenic	2 (6.1)	3 (11.5)	
Double-agent chemotherapy plus antiangiogenic	6 (18.2)	8 (30.8)	
Single-agent chemotherapy plus antiangiogenic	4 (12.1)	3 (11.5)	

ICI, immune checkpoint inhibitor; ECOG, Eastern Cooperative Oncology Group; RECIST, Response Evaluation Criteria in Solid Tumors.

### Efficacy of continuing anti- programmed cell death 1 therapy beyond progress

Efficacy data were assessable in all these 59 patients. The median follow-up time was 27.0 (minimum follow-up, 5.3 months; maximum follow-up, 47.0 months).

**Figure 1:**
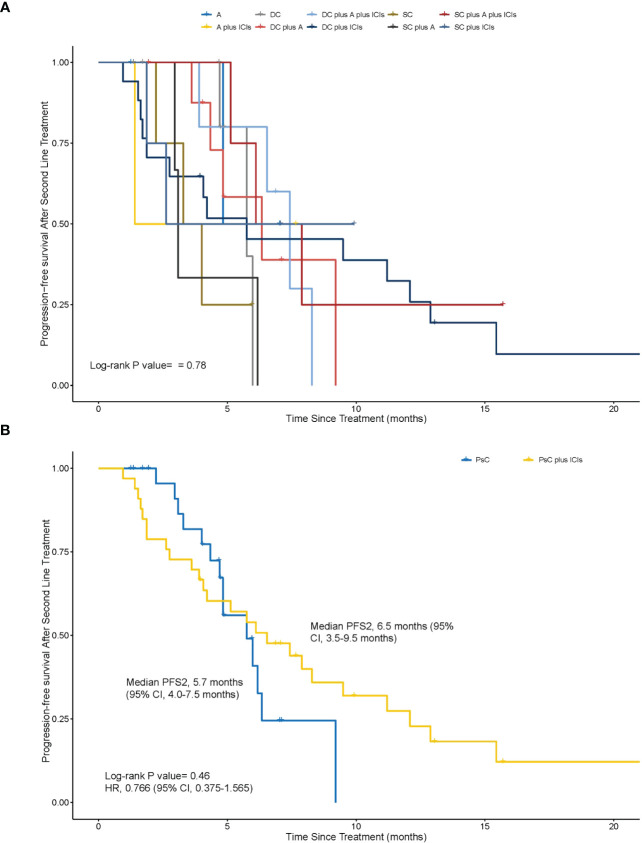
Kaplan–Meier estimates of progression-free survival in the second line (PFS2). **(A)** The medium PFS2 was 4.83 months in groups of antiangiogenic **(A)**, 1.41 months in groups of antiangiogenic plus immune checkpoint inhibitors (A plus ICIs), 5.75 months in groups of double-agent chemotherapy (DC), 6.34 months in groups of DC plus A, 7.43 months in groups of DC plus A plus ICIs, 5.75 months in groups of DC plus ICIs, 3.65 months in groups of single-agent chemotherapy (SC), 3.09 months in groups of SC plus A, 7.00 months in groups of SC plus A plus ICIs, and 2.63 months in groups of SC plus ICIs (*p* = 0.78). **(B)** PFS2 between the physician-decided second-line regimen plus ICI (PsC plus ICI) group and the physician-decided second-line regimen (PsC group) group in the second line (6.54 months vs. 5.74 months, *P* = 0.46, HR = 0.766, 95% confidence interval, 0.375–1.565).

The medium PFS2 was 4.83 months in groups of A, 1.41 months in groups of A plus ICIs, 5.75 months in groups of DC, 6.34 months in groups of DC plus A, 7.43 months in groups of DC plus A plus ICIs, 5.75 months in groups of DC plus ICIs, 3.65 months in groups of SC, 3.09 months in groups of SC plus A, 7.00 months in groups of SC plus A plus ICIs, and 2.63 months in groups of SC plus ICIs (**Figure 1A**). These groups were divided into two, the PsC plus ICIs group and the PsC group. The median PFS2 was 6.5 months (95% CI, 3.5–9.5 months) in the PsC plus ICIs group and 5.7 months (95% CI, 4.0–7.5 months) in the PsC group (HR = 0.766, 95% CI, 0.375–1.565; *p* = 0.46) (**Figure 1B**). The 8-month PFS2 rate was 30.3% (95% CI, 15.6%–48.7%) in the PsC plus ICIs group and 3.8% (95% CI, 0.1%–19.6%; *p* = 0.016) in the PsC group. For the overall tumor response in the second line, as shown in **Table 3**, the ORR was 18.2% and 19.2% and the DCR was 78.8% and 84.6% in the PsC plus ICIs group and the PsC group, respectively.

**Table 3:** Overall tumor response and efficacy in the second line.

Overall tumor response	PsC plus ICIs group (*N* = 33)	PsC group (*N* = 26)
Partial response, *n* (%)	6 (18.2)	5 (19.2)
Stable disease, *n* (%)	20 (60.6)	17 (65.4)
Progress disease, *n* (%)	7 (21.2)	2 (7.7)
Not evaluable	0 (0.0)	2 (7.7)
Objective response rate (CR + PR)	6 (18.2)	5 (19.2)
Disease control rate (CR + PR + SD)	26 (78.8)	22 (84.6)

**Figure 2:**
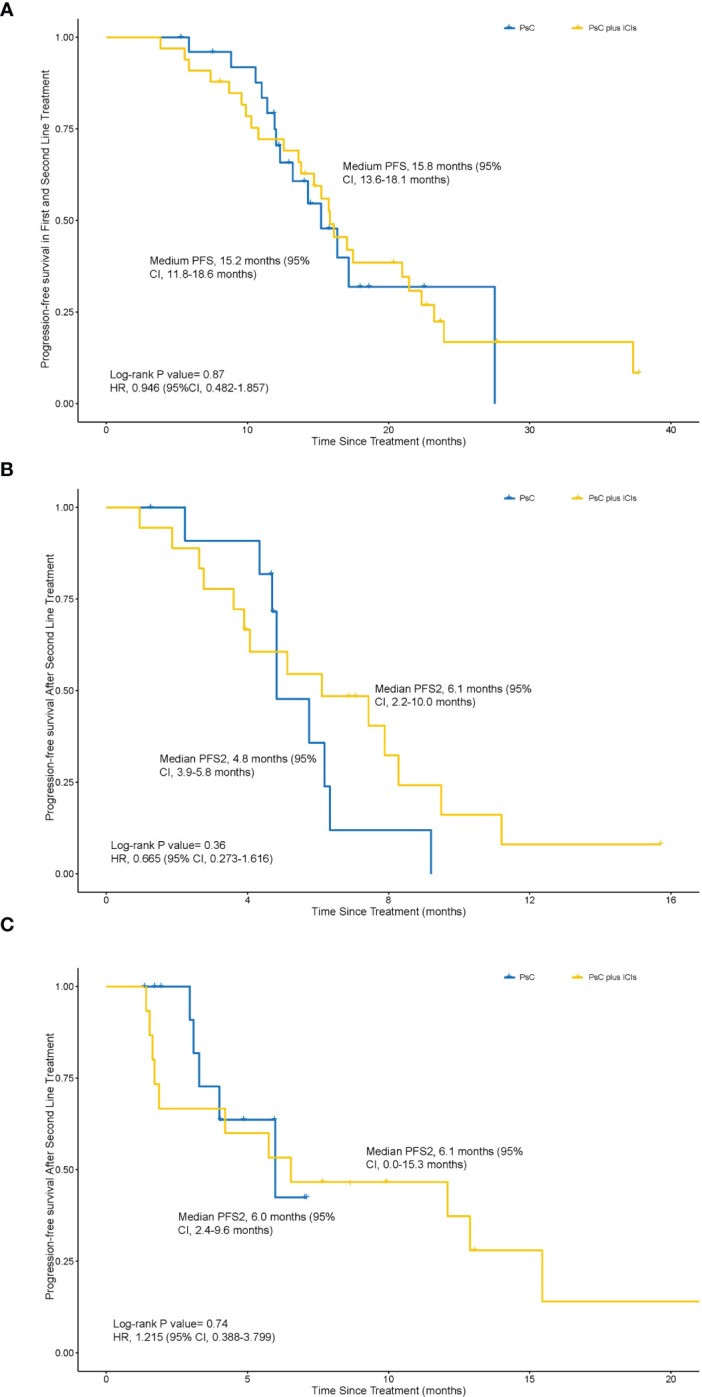
Kaplan–Meier estimates of progression-free survival in both the first line and second line. **(A)**, PFS1 + 2 between physician-decided second-line regimen (PsC group) group and physician-decided second-line regimen plus ICI (PsC plus ICI) group (15.2 months vs 15.8 months, *p* = 0.87, HR = 0.946, 95% CI, 0.482–1.857). **(B)** PFS2 in patients whose PFS1 was less than 8 months between the PsC group and the PsC plus ICI group in the second line (4.8 months vs 6.1 months, *p* = 0.36, HR = 0.665, 95% CI, 0.273–1.616). **(C)** PFS2 among patients whose PFS1 was more than 8 months between the PsC group and the PsC plus ICI group in the second line (6.0 months vs 6.1 months, *p* = 0.74, HR = 1.215, 95% CI, 0.388–3.799).

The medium PFS1 + 2 was 15.8 months (95% CI, 13.6–18.1 months) in the PsC plus ICIs group and 15.2 months (95% CI, 11.8–18.6 months) in the PsC group (HR = 0.946, 95% CI, 0.482–1.857; *p* = 0.87) (**Figure 2A**). The 18-month PFS1 + 2 rate was 33.3% (95% CI, 18.0%–51.8%) in the PsC plus ICIs group and 11.5% (95% CI, 2.4%–30.2%; *p* = 0.068) in the PsC group. The number of PFS1 is equal to 8 months as a cutoff value to determine whether patients benefit from the first line. There are no significant baseline characteristics between the PsC plus ICIs group and the PsC group (**Tables S3**, **S3**). In the group of PFS1 less than 8 months, the medium PFS2 was 6.1 months (95% CI, 2.2–10.0 months) in PsC plus ICIs group treatment and 4.8 months (95% CI, 3.9–5.8 months) in the PsC group (HR = 0.665, 95% CI, 0.273–1.616; *p* = 0.36) (**Figure 2B**). In the group of PFS1 more than 8 months, the medium PFS2 was 6.1 months (95% CI, 0.0–15.3 months) in the PsC plus ICIs group and 6.0 months (95% CI, 2.4–9.6 months) in the PsC group (HR = 1.215, 95% CI, 0.388–3.799; *p* = 0.74) (**Figure 2C**).

**Figure 3:**
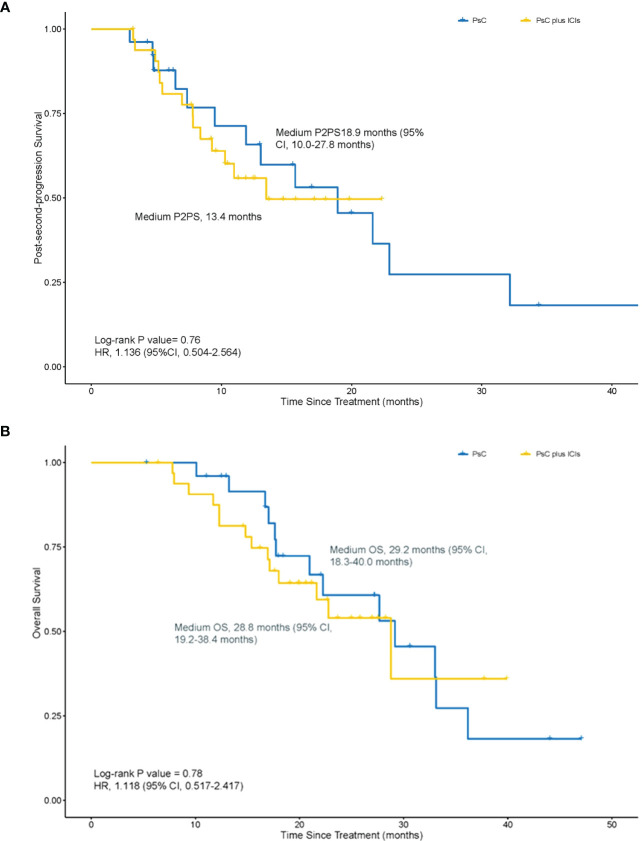
Kaplan–Meier estimates of post-second-progression survival (P2PS) and overall survival (OS). **(A)** P2PS between the PsC group and the PsC plus ICI group (18.9 months vs 13.4 months, p = 0.76, HR = 1.136, 95% CI, 0.504–2.564). **(B)** OS between the PsC group and the PsC plus ICI group (29.2 months vs. 28.9 months, p = 0.78, HR = 1.118, 95% CI, 0.517–2.417).

For all enrolled patients, the medium P2PS was 13.4 months in the PsC plus ICIs group and 18.9 months (95% CI, 10.0–27.8 months) in the PsC group (HR = 1.136, 95% CI, 0.504–2.564; *p* = 0.76) (**Figure 3A**). The medium OS was 28.8 months (95% CI, 19.2–38.4 months) in the PsC plus ICIs group and 29.2 months (95% CI, 18.3–40.0 months) in the PsC group (HR = 1.118, 95% CI, 0.517–2.417; *p* = 0.78) (**Figure 3B**).

Cox regression analysis, incorporating age, gender, smoking history, performance status, stage, histology, PFS1, and treatment regimen, further identified that PsC plus ICIs is not an independent prognostic factor of improved PFS2 and OS compared with PsC alone (HR = 0.635, 95% CI: 0.290–1.392, *p* = 0.257, **Table 4**; HR =1.116, 95% CI: 0.499–2.495, *p* = 0.790, **Table 5**).

**Table 4:** Univariate and multivariate Cox regression analyses of clinical parameters on progression-free survival after second-line treatment.

Variable	Category	Univariate analysis		Multivariate analysis	
		*P*	HR (95% CI)	*P*	HR (95% CI)
**Age**	<60 vs. ≥60	0.774	0.910 (0.478-1.732)	0.180	0.602 (0.286-1.266)
**Gender**	Female vs. Male	0.647	1.247 (0.486-3.200)	0.983	0.987 (0.282-3.459)
**Smoke**	Never vs. Smoker	0.241	1.491 (0.764-2.910)	0.041	2.865 (1.047-7.842)
**Performance Status (ECOG)**	0 vs. ≥1	0.114	1.738 (0.876-3.448)	0.176	1.646 (0.800-3.387)
**Histology**	Squamous vs. Non-Squamous	0.822	0.927 (0.479-1.794)	0.494	0.754 (0.337-1.691)
**Stage**	IIIC vs. IV	0.407	1.552 (0.549-4.386)	0.300	1.787 (0.596-5.358)
**First-line progression-free survival, mo**	<8 vs. ≥8	0.213	0.661 (0.344-1.268)	0.124	0.572 (0.280-1.166)
**Treatment regimen**	PsC vs. PsC plus ICIs	0.465	0.766 (0.375-1.565)	0.257	0.635 (0.290-1.392)

Bold values indicate P < 0.05; CI, confidence interval; HR, hazard ratio.

**Table 5:** Univariate and multivariate Cox regression analyses of clinical parameters on overall survival.

Variable	Category	Univariate analysis		Multivariate analysis	
		*P*	HR (95% CI)	*P*	HR (95% CI)
**Age**	<60 vs. ≥60	0.081	2.090 (0.912–4.787)	0.016	4.637 (1.336–16.094)
**Gender**	Female vs. Male	0.625	1.349 (0.405–4.492)	0.052	5.109 (0.988–26.431)
**Smoke**	Never vs. Smoker	0.709	1.158 (0.536–2.499)	0.274	0.439 (0.101–1.916)
**Performance Status (ECOG)**	0 vs. ≥1	0.736	0.865 (0.372–2.012)	0.154	00.483 (0.177–1.313)
**Histology**	Squamous vs. Non-squamous	0.884	1.058 (0.492–2.276)	0.836	0.882 (0.270–2.889)
**Stage**	IIIC vs. IV	0.899	0.933 (0.318–2.733)	0.620	0.725 (0.207–2.553)
**First-line progression-free survival, mo**	<8 vs. ≥8	0.010	0.355 (0.162–0.782)	0.001	0.211 (0.083–0.538)
**Treatment regimen**	PsC vs. PsC plus ICIs	0.777	1.118 (0.517–2.417)	0.790	1.116 (0.499–2.495)

Bold values indicate P < 0.05; CI, confidence interval; HR, hazard ratio.

### Tolerability

The detailed adverse events in the second line are presented in **Table S4**. During anti-PD-1 treatment in the first line, grade 2−4 treatment–related toxicities leading to permanent discontinuation of immunotherapy occurred in three (5.1%) of all patients. The three cases were immune-related hypophysitis, adrenal insufficiency, and pneumonia, respectively. Moreover, two patients (3.4%) temporarily stopped receiving anti-PD-1 treatment due to immune-related hepatitis and pneumonia. In the group continuing anti-PD-1 therapy beyond progress, only one patient (3.3%) permanently stopped receiving immunotherapy due to immune-related enteritis.

## Discussion

This retrospective analysis suggests that most patients with advanced NSCLC who continued ICI treatment beyond RECIST v1.1–defined progression may not derive apparent clinical benefit. We identified that there is no difference in PFS2, PFS1 + 2, P2PS, and OS between the PsC plus ICIs group and the PsC group, although the 8-month PFS2 rate is higher in the PsC plus ICIs group. It is noteworthy that we saw a negative clinical benefit of continuing ICI treatment after disease progression.

So many second-line phase III studies have been done in the past decade. Before the use of immunotherapy, except the TAX 317 trail (20), docetaxel versus best supportive care, none of these has shown a significant improvement in OS. Studies of targeted agents in combination with standard second-line therapy, including nintedanib (21), ramucirumab (22), and bevacizumab (23), have shown a significant improvement in PFS and OS. After the use of immunotherapy, in patients who had progressed after one previous PBC, pembrolizumab, nivolumab, and atezolizumab demonstrated superiority over docetaxel in NSCLC (11–14). However, it still lacks prospective clinical trials to confirm the optimal regimens used after chemoimmunotherapy beyond PD. In this current study, we observed in the real world, antiangiogenic combined with ICIs get the worst PFS2 (1.41 months), even worse than treated along with antiangiogenic (PFS2, 4.83 months). Up to now, bevacizumab is the only antiangiogenic agent approved for first-line treatment of NSCLC (24). Also, results from the IMPOWER 150 study showing the efficacy of a combination of paclitaxel, carboplatin, bevacizumab, and atezolizumab may enhance treatment outcomes and lead to better survival (25), although the quadruplet is not yet approved by the FDA. In our study, the combined treatment, including chemotherapy plus antiangiogenic plus ICIs, either single-agent chemotherapy or double-agent chemotherapy (PFS2, 7.00 and 7.43 months, respectively), confers a survival benefit more than others. Recently, bispecific antibody (bsAbs) research around the world has undergone great changes (26). However, no clinical trials have allowed NSCLC patients who have received ICIs in the past. Therefore, our research is enlightening and shows the potential for clinical practice and future research to explore the detailed treatment option after the failure of chemoimmunotherapy.

There are limited options for patients who have PD after chemoimmunotherapy, and the survival outcomes are poor. Whether ICI should be sustained is an important, unanswered question, as recently described in multiple other tumor types (27). In our study, the 8-month PFS2 rate was higher in the PsC plus ICIs group than that in the PsC group, although there is no significance in the median PFS2. Furthermore, there are no significant differences in PFS1 + 2, and the 18-month PFS1 + 2 rate, as in P2PS and OS. In addition, the ORR and DCR were also almost the same in both groups. To date, interest in the potential role of ICIs is broad. P2PS, a term implying continued benefit in OS after PD, originally applied to the continuation of EGFR inhibitor therapy, provides a rationale for continued ICI therapy (28). Analyses of ICI treatment beyond progression of anti-PD-1/PD-L1 in metastatic urothelial carcinoma, renal cell carcinoma, melanoma, and metastatic NSCLC have been reported (18, 19, 29, 30). These studies suggest that continued ICI treatment benefited some patients in terms of tumor reduction, as well as longer median OS compared to untreated patients beyond progression. However, in our study, we find that few patients may benefit from continued ICI treatment. We regard 8 months in PFS1 as a cutoff value to determine whether patients benefit from the first line using ICIs. Regardless of whether patients benefit from first-line chemoimmunotherapy, it does not make a clinical benefit for them to continue immunotherapy in the second line. Thus, our study challenges the previously reported efficacy of continuing immunotherapy beyond PD.

With regard to the safety of ICI treatment beyond PD, continuing ICIs were well tolerated, and no new or unexpected AEs observed in our study. No immunotherapy-related toxicities occurred in the discontinued ICI treatment group. Only one patient permanently stopped receiving immunotherapy due to immune-related enteritis in the continued ICI treatment group. The patient totally received 22 cycles of pembrolizumab combined with chemotherapy both in the first and second lines. The patient presented with severe diarrhea and was considered for immunotherapy-related enteritis. As reported in the efficacy of ICIs in patients with other tumors treated beyond progression (19, 27), there was no significant difference in the rate of treatment-related adverse events between patients who continued ICIs and those who did not. Attention also needs to be paid to new or unexpected AEs due to continued treatment, which may be associated with risks reported in metastatic renal cell carcinoma (31). In this article, we observed that patients with advanced NSCLC may be well tolerated continuing ICI treatment beyond disease progression.

In spite of the fact that our analysis provides insights into the extended use of ICIs beyond disease progression, interpretation of these results is limited by a number of factors, including the relatively small number of patients; the use of retrospective data; the unused response evaluation criteria for immunotherapy, such as irRC, iRECIST, and irRECIST; and the investigators’ selection of patients for extended treatment based on factors that have not been systematically explored. However, the study also has major strengths, in particular the multiple treatments in line with the real world and the well-balanced characteristics of each group. Future studies should examine outcomes and safety in patients’ continued ICIs beyond disease progression in large randomized prospective cohorts and identify which characteristics patients are likely to benefit most from this treatment.

## Conclusion

Our analysis shows that continued anti-PD-1 immunotherapy beyond initial progression may not improve clinical benefit for patients with NSCLC, and it is a safety profile consistent with that observed in patients who discontinued ICI treatment. In light of the new data on immunotherapy, it will be interesting to see how patients who progress to chemoimmunotherapy in the first line will receive further treatment options. Our study can serve as an important reference value for such studies.

## Data availability statement

The raw data supporting the conclusions of this article will be made available by the authors, without undue reservation.

## Ethics statement

This study protocol is approved by the Sun Yat-Sen University Cancer Center Institutional Review Board. Written informed consent from the patients/participants OR patients/participants legal guardian/next of kin was not required to participate in this study in accordance with the national legislation and the institutional requirements.

## Author contributions

SH and LZ contributed to the study design. YW, SF, and XZ contributed to the sample collection and patient data management. SF and XZ analyzed the samples. YW drafted the manuscript. SH revised the manuscript. All authors read and approved the final manuscript.
