# Supplementary material for: Continuation of anti-PD-1 therapy plus physician-choice treatment beyond first progression is not associated with clinical benefit in patients with advanced non-small cell lung cancer
Source: Front Immunol. 2023 May 29;14:1151385. doi: 10.3389/fimmu.2023.1151385 (PMC10258328; doi:10.3389/fimmu.2023.1151385)
Supplement: Supplementary file 1 [file Table_1.docx]

Table S1. Treatment in second line.

| Treatment (N=59) | NO. (%) |
| --- | --- |
| **Single-agent chemotherapy plus ICIs** |  |
| Albumin Paclitaxel plus ICIs | 3 (5.1) |
| Gemcitabine plus ICIs | 1 (1.7) |
| **Double-agent chemotherapy plus ICIs** |  |
| Albumin Paclitaxel plus Platinum plus ICIs | 9 (15.2) |
| Gemcitabine plus Platinum plus ICIs | 5 (8.5) |
| Pemetrexed plus Platinum plus ICIs | 3 (5.1) |
| **Antiangiogenic plus ICIs** |  |
| Anlotinib plus ICIs | 1 (1.7) |
| Bevacizumab plus ICIs | 1(1.7) |
| **Double-agent chemotherapy plus Antiangiogenic plus ICIs** |  |
| Albumin Paclitaxel plus Platinum plus Antiangiogenic plus ICIs | 4 (6.8) |
| Pemetrexed plus Platinum plus Antiangiogenic plus ICIs | 1 (1.7) |
| **Single-agent chemotherapy plus Antiangiogenic** |  |
| Albumin Paclitaxel plus Antiangiogenic | 2 (3.4) |
| Platinum plus Antiangiogenic | 1 (1.7) |
| **Double-agent chemotherapy plus Antiangiogenic** |  |
| Albumin Paclitaxel plus Platinum plus Antiangiogenic | 8 (13.6) |
| Pemetrexed plus Platinum plus Antiangiogenic | 1 (1.7) |
| **Single-agent chemotherapy** |  |
| S-1 | 1 (1.7) |
| Albumin Paclitaxel | 3 (5.1) |
| **Double-agent chemotherapy** |  |
| Gemcitabine plus Platinum | 5 (8.5) |
| Albumin Paclitaxel plus Platinum | 2 (3.4) |
| Etoposide plus Platinum | 1 (1.7) |
| **Antiangiogenic** |  |
| Anlotinib | 3 (5.1) |
| **Single-agent chemotherapy plus Antiangiogenic plus ICIs** |  |
| Gemcitabine plus Antiangiogenic plus ICIs | 1 (1.7) |
| Albumin Paclitaxel plus Antiangiogenic plus ICIs | 2 (1.7) |
| VP16 plus Antiangiogenic plus ICIs | 1 (1.7) |

Abbreviation: ICI, immune checkpoint inhibitor.

Table S2. Baseline characteristics of the included patients whose PFS1 <8 months treated with or without ICIs.

| Characteristics | PsC plus ICIs group  (N=18) | PsC group  (N=12) | *P* |
| --- | --- | --- | --- |
| **Age** |  |  | 0.509 |
| Median (range) | 60 (31-72) | 59.5 (33-70) |  |
| **Sex** |  |  | 0.660 |
| female | 3 (50.0) | 3 (50.0) |  |
| Male | 15 (62.5) | 9 (37.5) |  |
| **ECOG performance** |  |  | 0.531 |
| 0 | 13 (65.0) | 7 (35.0) |  |
| 1 | 5 (55.6) | 4 (44.4) |  |
| 2-3 | 0 (0.0) | 1 (100.0) |  |
| **Smoking status** |  |  | 0.457 |
| Never smoker | 7 (50.0) | 7 (50.0) |  |
| Current or former smoker | 11 (68.8) | 5 (31.3) |  |
| **Pathology** |  |  | 1.000 |
| Squamous cell carcinoma | 11 (61.1) | 7(38.9) |  |
| Non-Squamous cell carcinoma | 7 (58.3) | 5 (41.7) |  |
| **Stage** |  |  | 1.000 |
| IIIC | 3 (60.0) | 2 (40.0) |  |
| IV | 15 (60.0) | 10 (40.0) |  |
| **No. of metastatic sites** |  |  | 0.266 |
| 0-1 | 13 (68.4) | 6 (31.6) |  |
| ≥2 | 5 (45.5) | 6 (54.5) |  |
| **RECIST response in first line** |  |  | 0.572 |
| PR | 8 (57.1) | 6 (42.9) |  |
| SD | 9 (69.2) | 4 (30.8) |  |
| PD | 1 (33.3) | 2 (66.7) |  |
| **Treatment in second line** |  |  | 0.430 |
| Single-agent chemotherapy | 3 (75.0) | 1 (25.0) |  |
| Double-agent chemotherapy | 6 (60.0) | 4 (40.0) |  |
| Antiangiogenic | 0 (0.0) | 2 (100.0) |  |
| Double-agent chemotherapy plus Antiangiogenic | 5 (55.6) | 4 (44.4) |  |
| Single-agent chemotherapy plus Antiangiogenic | 4 (80.0) | 1 (20.0) |  |

Abbreviation: ICI, immune checkpoint inhibitor; ECOG, Eastern Cooperative Oncology Group; RECIST, Response Evaluation Criteria in Solid Tumors.

Table S3. Baseline characteristics of the included patients whose PFS1 ≥8 months treated with or without ICIs.

| Characteristics | PsC plus ICIs group  (N=15) | PsC group  (N=14) | *P* |
| --- | --- | --- | --- |
| **Age** |  |  | 0.299 |
| Median (range) | 58 (31-74) | 64 (42-68) |  |
| **Sex** |  |  | 0.224 |
| female | 0 (0.0) | 2 (100.0) |  |
| Male | 15 (55.6) | 12 (44.4) |  |
| **ECOG performance** |  |  | 0.489 |
| 0 | 10 (52.6) | 9 (47.4) |  |
| 1 | 2 (33.3) | 4 (66.7) |  |
| 2-3 | 3 (75.0) | 1 (25.0) |  |
| **Smoking status** |  |  | 1.000 |
| Never smoker | 6 (54.5) | 5 (45.5) |  |
| Current or former smoker | 9 (50.0) | 9 (50.0) |  |
| **Pathology** |  |  | 0.715 |
| Squamous cell carcinoma | 6 (46.2) | 7 (53.8) |  |
| Non-Squamous cell carcinoma | 9 (56.3) | 7 (43.8) |  |
| **Stage** |  |  | 0.598 |
| IIIC | 1 (33.3) | 2 (66.7) |  |
| IV | 14 (53.8) | 12 (46.2) |  |
| **No. of metastatic sites** |  |  | 0.390 |
| 0-1 | 10 (45.5) | 12 (54.5) |  |
| ≥2 | 5 (74.1) | 2 (28.6) |  |
| **RECIST response in first line** |  |  | 0.091 |
| PR | 5 (35.7) | 9 (64.3) |  |
| SD | 10 (71.4) | 4 (28.6) |  |
| PD | 0 (0.0) | 1 (100.0) |  |
| **Treatment in second line** |  |  | 0.067 |
| Single-agent chemotherapy | 1 (25.0) | 3 (75.0) |  |
| Double-agent chemotherapy | 11 (73.3) | 4 (26.7) |  |
| Antiangiogenic | 2 (66.7) | 1 (33.3) |  |
| Double-agent chemotherapy plus Antiangiogenic | 1 (20.0) | 4 (80.0) |  |
| Single-agent chemotherapy plus Antiangiogenic | 0 (0.0) | 2 (100.0) |  |

Abbreviation: ICI, immune checkpoint inhibitor; ECOG, Eastern Cooperative Oncology Group; RECIST, Response Evaluation Criteria in Solid Tumors.

Table S4. Overview of Main Adverse Events in second line.

| Adverse Events, N (%) | PsC plus ICIs group  (N=33) | | PsC group  (N=26) | |
| --- | --- | --- | --- | --- |
|  | Any grade | Grade 3/4 | Any grade | Grade 3/4 |
| Leukopenia | 2 (7.7) | 2 (7.7) | 3 (9.1) | 1(3.0) |
| Neutropenia | 1 (3.8) | 0 (0.0) | 3 (9.1) | 1(3.0) |
| Thrombopenia | 1 (3.8) | 1 (3.8) | 3 (9.1) | 1(3.0) |
| Anemia | 2 (7.7) | 0 (0.0) | 1(3.0) | 0 (0.0) |
| Fatigue | 1 (3.8) | 0 (0.0) | 1(3.0) | 0 (0.0) |
| Anorexia | 2 (7.7) | 0 (0.0) | 1(3.0) | 0 (0.0) |
| Nausea | 1 (3.8) | 0 (0.0) | 3 (9.1) | 0 (0.0) |
| Vomiting | 1 (3.8) | 0 (0.0) | 2 (6.1) | 0 (0.0) |
| Diarrhea | 0 (0.0) | 0 (0.0) | 2 (6.1) | 0 (0.0) |
| Peripheral sensory neuropathy | 2 (7.7) | 0 (0.0) | 2 (6.1) | 0 (0.0) |
| Immune-related enteritis | 1 (3.8) | 0 (0.0) | 0 (0.0) | 0 (0.0) |

Adverse events occurred in at least 20% of patients across all cycles.
